# Vaginal Herpes Zoster While Under Treatment for Relapsing-Remitting Multiple Sclerosis With Diroximel Fumarate

**DOI:** 10.7759/cureus.40689

**Published:** 2023-06-20

**Authors:** Sedat Gül, Adeenah F Ahmed, Corey McGraw

**Affiliations:** 1 Neurology, State University of New York Upstate Medical University, Syracuse, USA

**Keywords:** vaginal fungal infections, varicella-zoster virus, vumerity, diroximel fumarate, relapsing-remitting multiple sclerosis (rrms)

## Abstract

Multiple sclerosis (MS) is an autoimmune demyelinating disorder that disproportionately affects middle-aged women, and is capable of resulting in severe disability. However, the use of disease-modifying therapies has profoundly contributed to the improvement in the morbidity of the disorder. Diroximel fumarate (DRF) is a second-generation drug that has seen success in the treatment of relapsing-remitting MS (RRMS). While its relatively mild side effects of gastrointestinal discomfort are known, the less common complications are often missed in clinical settings. This includes a resulting susceptibility to opportunistic infections. In this case report, we describe a patient who experienced lymphopenia, recurrent yeast infections, and labial shingles while on the medication. This case highlights the side effects and the rare complications of the immunomodulator, DRF.

## Introduction

Multiple Sclerosis (MS) is an autoimmune demyelinating disorder that affects the central nervous system (CNS). It is typically diagnosed between the ages of 20 and 40 years, and more commonly in female patients [1]. Subtypes of MS include relapsing forms, primary progressive, and secondary progressive [2]. Several disease-modifying therapies exist for the treatment of MS, especially for the relapsing-remitting form. Fumaric acid esters, including the first-generation dimethyl fumarate (DMF) and second-generation diroximel fumarate (DRF), are being used extensively. These disease-modifying therapies can decrease the number of relapses for patients with relapsing forms [3]. Diroximel fumarate is a next-generation drug utilized for MS that has been tolerated well and is noted to have good adherence due to its oral formation (versus injectable or infusion therapy options) [4]. Possible side effects, especially within the first month of use, include gastrointestinal symptoms such as diarrhea and nausea, and flushing; however, these effects are typically not associated with decreased use of the medication [5,6]. Although less common, lymphopenia, or a low white blood cell count, may also occur [7].

Varicella zoster virus (VZV) is a DNA virus. In childhood, infection with VZV is colloquially known as chickenpox. This virus is capable of residing within the CNS to establish latency and then result in symptoms later on in life secondary to an immunocompromised state. This infection results in a typical clinical presentation consisting of a painful, vesicular rash along a particular dermatome unilaterally. This clinical condition is known as herpes zoster (HZ), or shingles colloquially. As mentioned, shingles can arise during a state of immunocompromisation. This includes patients with advanced or untreated HIV infection, primary immunodeficiency syndromes, and active treatment with high-dose corticosteroids or other immunomodulatory agents [8]. By definition, immunosuppressants can increase the risk of opportunistic infections, such as viral or fungal infections. For a practicing neurologist, knowledge of possible side effects and possible presentations of infections while placing patients on disease-modifying therapies is imperative.

Here, we describe the case of a 45-year-old woman with relapsing-remitting MS (RRMS) who has a history of childhood encephalitis due to a chickenpox infection. During her course of DRF treatment, she experienced lymphopenia, recurrent yeast infections, and labial shingles.

## Case presentation

A 45-year-old woman with RRMS presented to the clinic. Her past medical history includes non-alcoholic fatty liver disease, type 2 diabetes mellitus, and childhood encephalitis during chicken pox. She had one episode of facial numbness and one episode of right lower extremity paresthesia in the past eight years due to MS flares. She was started on DMF. Within the first year of therapy, she was diagnosed with endometrial cancer and underwent a hysterectomy. Six months after the hysterectomy, the patient began to experience gastrointestinal side effects of DMF hence the medication was discontinued. The patient stayed on DMF for 20 months in total. Due to the recent diagnosis of endometrial cancer that led to a hysterectomy while the patient was on DMF, transaminitis, and the patient’s preference of not having injections, DRF was selected as the new regimen. The patient used the medication for two months continuously except for a period of 10 days when she had a tooth abscess. At the end of two months, she developed a painful rash in her genital area. Skin swab samples were obtained, and polymerase chain reaction (PCR) confirmed VZV DNA, and the patient was diagnosed with vaginal shingles. Symptoms improved after a seven-day course of valacyclovir treatment and the patient continued taking DRF. However, she never achieved full symptomatic recovery of the genital rash, and residual pain persisted. Due to unresolved symptoms such as rash, erythema, pruritus, and pain, an additional workup was performed that included fungal cultures. The patient was diagnosed with vaginal candida albicans infection by her gynecologist. At that time, the patient had mild lymphopenia (848 cell/uL and 383 CD4 cell/uL) (Table 1) and borderline low IgG level (691 mg/dL). Due to the recurrent vaginal infections, DRF was stopped and glatiramer acetate was started. The patient’s vaginal infections resolved one month after stopping DRF. The vaginal lesions resolved completely, and she has not experienced another MS flare.

**Table 1:** Lymphocyte subsets obtained at the time of herpes zoster diagnosis NK: Natural killer, CD: Cluster of differentiation

Parameters	Reference	Patient’s value
Lymphocyte count	1200-4000 cells/uL	848
CD19 % B cells	6-%23%	14
CD19 absolute B cells	91-610 cells/uL	121
CD3% mature T cells	62%-87%	64
CD3% absolute T cells	570-2400 cells/uL	543
CD4% helper/inducer	21%-64%	47
CD4 absolute count	430-1800 cells/uL	383
CD8% suppress/cytotoxic	15%-46%	15
CD8 absolute count	210-2100 cells/uL	124
CD56/16% NK cells	4%-26%	21
CD56/16 NK cell absolute	78-470 cells/uL	187
CD4/CD8 ratio	0.8-3.9	3.1

## Discussion

Multiple sclerosis is a debilitating disease that afflicts approximately 1 million people in the United States [9]. Immunomodulators have contributed tremendously to the treatment of those with MS, preventing relapses and increasing their quality of life. Conversely, side effects and rare complications of such therapies should be considered. An immunocompromised state yields opportunities for infections that may result in further debilitation.

Genital HZ is a well-documented entity, especially in immunocompromised patients [10]. Genital dermatomes are involved in up to 2% of all HZ cases, which makes it an underrecognized presentation [11]. Treatment is usually acyclovir or valacyclovir and an additional regimen for pain [12]. Most patients recover in several weeks, with the rash being resolved. Pain may persist after the resolution of skin manifestations. In our case, the patient’s rash didn’t completely resolve, and she had residual symptoms such as pruritus and erythema. Further investigation revealed a fungal infection. Herpes zoster with overlapping fungal infection was not reported in the literature to the best of our knowledge. This is an important point to be aware of, especially for patients who take immunomodulators. Also, our patient switched to another immunomodulator, namely glatiramer acetate. However, she did not experience a recurrence of her symptoms. It is unclear why she experienced recurrent infections while on DRF and switching the therapy benefited her.

## Conclusions

Patients on immunomodulators have an increased risk of suffering from opportunistic infections, sometimes disguised as common pathogens, especially among the young and healthy population. Here, we described a young female patient with RRMS who suffered from recurrent vaginal infections, VZV, and a fungal infection when she was on DRF. Her infections resolved once the medication was stopped. We highlighted a previously unreported side effect of DRF that should be taken into account for female patients, i.e., recurrent vaginal infections. Also, for the first time in the literature, we reported the case of an overlapping fungal infection in the setting of HZ in a patient with MS.
